# Surgical Management of Neurogenic Sphincter Incompetence in Children

**DOI:** 10.3389/fped.2019.00097

**Published:** 2019-03-26

**Authors:** Barbara M. Ludwikowski, Jan-Christoph Bieda, Anja Lingnau, Ricardo González

**Affiliations:** ^1^Pediatric Surgery and Urology, Kinder- und Jugendkrankenhaus Auf der Bult, Hanover, Germany; ^2^Department of Urology, Charité Medical University of Berlin, Berlin, Germany

**Keywords:** urinary incontience, neurogenic bladder, bladder neck closure, bladder neck reconstruction, urethral slings, artificial sphincters, injection of bulking agents, children

## Abstract

We report on the results of a literature review regarding the indications and results of operations to increase bladder outlet resistance to achieve dryness in children with neurogenic sphincter incompetence (NSBD). The relative advantages and disadvantages of injection of bulking agents, periurethral slings, bladder neck reconfiguration, artificial sphincters, and bladder neck closure based on a literature review and our combined clinical experience are discussed. Based on this review and our experience, we propose that periurethral injection of bulking agents is not justified as a primary treatment. Likewise, operations that reconfigure the bladder neck are not very useful since most patients also require bladder augmentation and an abdominal catheterizable channel. Bladder neck slings with autologous tissues are effective, mostly in females bur in the majority of patients a bladder augmentation is necessary. There is a role also for implantation of artificial urinary sphincters but when done as an isolated procedure, close monitoring to detect possible detrusor changes is needed. Bladder neck closure is an effective measure when other methods have failed.

## Introduction

Urinary incontinence in children with neurogenic bladder and sphincter dysfunction (NBSD) is common (1). NBSD can be congenital or acquired. The most frequent etiologies in children are spina bifida, sacral agenesis, other spinal malformations (such as those associated with anorectal malformations), spinal trauma, and iatrogenic surgical injuries.

The pathophysiology of urinary incontinence in children with neurogenic bladder dysfunction (NVD) is often complex and should be clearly defined before deciding on which therapeutic alternatives are most likely to succeed.

Urinary incontinence results from an imbalance between bladder storage pressures and bladder outlet resistance. The anamnesis, voiding diary and urodynamic studies help in arriving at the correct therapeutic decision.

**Figure 1:**
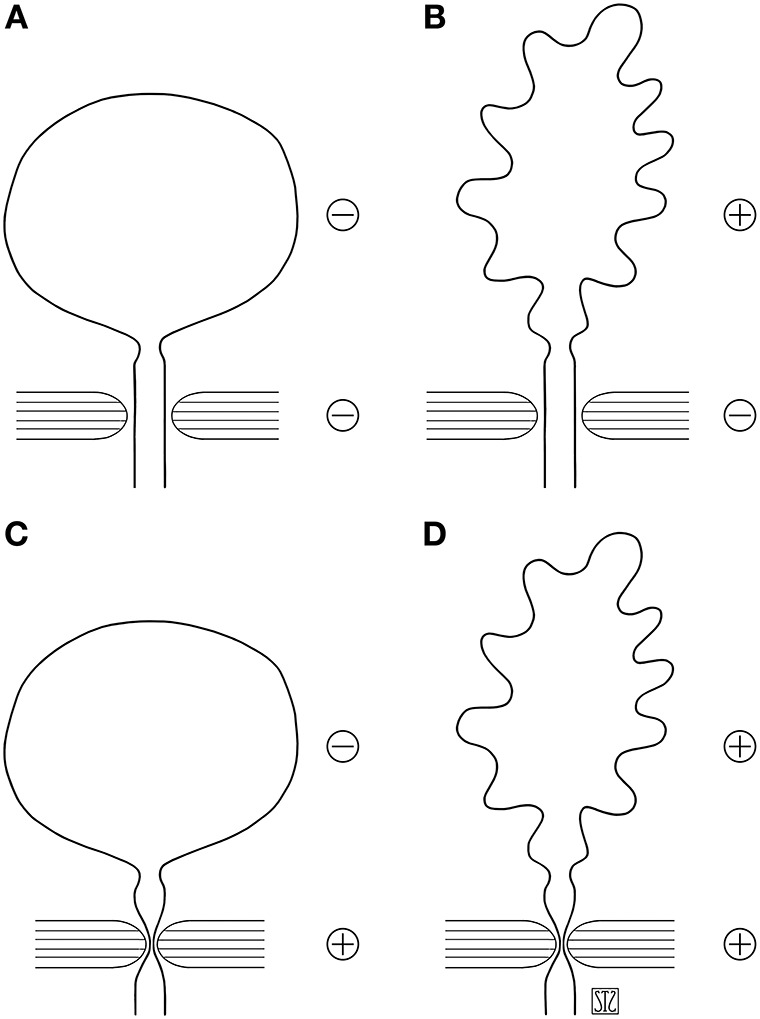
Schematic grouping of causes of neurogenic incontinence based on urodynamic findings [adapted from González and Guzmán (2)]. **(A)** Detrusor and sphincter mechanisms hypoactive. **(B)** Detrussor hyperactive or hypocompliant, sphincter hypoactive. **(C)** Detrusor hypoactive, sphincter hyperactive. **(D)** Detrusor and sphincter hyperactive (dyssynergic). Reproduced with permission from Dr. Quek.

The scheme proposed by J. M. Guzmán helps placing patients in one of four groups based on the information obtained by urodynamic studies and simplifies decision making (Figure 1) (3). Patients in groups A and B have low outlet resistance and require procedures to increase it, which are the object of this review.

In most children with NBSD successful therapy can almost always be equated to dryness rather that true continence although such distinctions are often not clear in the literature (4). If we accept that the definition of incontinence is the involuntary loss of urine, then continence should be the ability to voluntarily or involuntary avoid losing urine in the course of normal daily activities or during sleep. Of course, most patients with NBSD can seldom voluntarily control voiding and therefore it seems more appropriate to define a successful treatment in these cases as dryness, followed by the time period during which a patient is expected to be dry.

The backbone of treatment of NVD and NBSD is intermittent clean catheterization (5) since in most cases the bladder does not empty efficiently, particularly after procedures to increase outlet resistance and attain dryness have been performed. This includes patients who can occasionally void spontaneously with an implanted artificial sphincter.

Non-surgical treatments for incontinence in NBSD have been advocated by some (6) but are generally ineffective (7).

One problem in deciding which procedure is best for a given patient is the interpretation of the published literature which provides low levels of evidence and lacks uniformity in reporting results (8, 9). Therefore, it is difficult to reach solid conclusions from a review of this subject and personal experience and expert opinions are inevitably used in making clinical decisions.

The purpose of this review is to orient the interested clinician in this complex and often confusing topic. In this article we shall strive at objectivity and fairness but it must be recognized that lack of solid evidence in the literature (8) plus our long combined experience treating these patients may influenced our judgment. One of the authors participated in a review of the subject 18 years ago (10) and we will try to contrast the conclusions reached then with the conclusions reached in the present review.

## Materials and Methods

Review of the literature obtained by searching PubMed, Cochrane reviews and Google scholar under the words neurogenic urinary incontinence in children, artificial sphincter, sling, bladder neck reconstruction, and bulking agents among others. Abstracts and full text articles when available, in English, German, Spanish, French and Portuguese were reviewed. Abstracts that defined the criteria for patient selection, treatment employed, evaluation of outcomes and length of follow up form the basis for this review. Full text articles of the significant abstract were reviewed. Citations in the reviewed articles that were considered significant were included as well.

## Results

The results presented derive from the review of the papers obtained in the literature search.

Surgical procedures to increase bladder outlet resistance can be grouped in 4 categories: (1) Periurethral injection of bulking substances to exert external compression of the urethral lumen, (2) Procedures to reconfigure the bladder neck, (3) Bladder neck suspension and periurethral slings and, (4) Artificial sphincters and other prosthetic devices, and (5) Bladder neck closure.

### Periurethral Injection of Bulking Substances

Numerous substances have been injected trans- or periurethrally in hopes to increase passive outlet resistance and to increase the leak point pressure.

Probably the first substance used was polytetrafluoroethylene paste reported more than 40 years ago (11) including in children (12), however its use was discontinued following reports of potentially dangerous migration of the substance to remote organs including the brain (13). Other substances followed, among others bovine collagen, autologous fat, polydimethylsiloxane, autologous chondrocytes, stem cells and dextranomer/hyaluronic acid (Dx/HA). In this section we review the relevant literature available for the use of bulking agents in children.

In the last 20 years some retrospective or prospective non randomized studies were published.

Injection of Dx/HA was mostly performed retrograde transurethral. Antegrade injection through an appendicovesicostomy (Mitrofanoff channel) or a suprapubic access was preferred in selected cases to obtain better view (14, 15). A suprapubic catheter or a catheter through the catheterizable channel was left indwelling for 3 days to 2 weeks (14).

Some authors reported repeated injections to achieve dryness (15, 16) while others found no improvement after second injections and did not recommend it (14, 17).

Most of the series have short term follow up. At 6 months of follow up two thirds of the patients had improvements in dry intervals (18, 19) with a decrease of the success rate to 50% at a follow up of 12 months (18). At longer follow up injection of Dx/HA resulted in improvement in half of the patients with dryness in 40% in the series reporting the best results (16, 17, 20). Recurrence of incontinence was considered mainly a sign for bladder deterioration and should call for an urodynamic evaluation (20).

Dean et al. published better results in using an antegrade injection technique and leaving a suprapubic tube for 1 to 2 weeks. They treated 34 patients, 28 with the diagnosis of neurogenic bladder and 6 non-neurogenic sphincter incompetence. In 19 patients a mean follow up of 11.7 months was available. Fifteen of the 19 patients (79%) reported significant improvement of the incontinence after multiple injections (15).

Dx/HA injections were performed primary or secondary after failed sling procedures or bladder neck reconstruction After failed sling procedures dryness was gained in 7–25% of the patients (14, 17, 20). After bladder neck reconstruction in patients with neurogenic bladder Faure et al. reported a continence rate of 54% (21).

The studies showed that endoscopic injection in the bladder neck is safe with a low complication rate (16).

Unfortunately the outcome of the injection was not predictable by urodynamic parameters, the endoscopic technique or by the volume of injected volume (14, 20). Females had a significantly higher success rate of 69 vs. 38% (20).

Alova et al. found no difference in success of further surgical procedures (bladder neck reconfiguration, artificial sphincter or sling procedures) after failed endoscopic injections (22).

Endoscopic injection of the bladder neck can be combined with transurethral injection of botulinum toxin A in the detrusor to enlarge the bladder capacity. However, in one series 16 children required 54 injections of botulin toxin and 13 children 24 injections of Dx/Ha over a 4 year period to attain “social continence” (19).

The use of stem cells injected in the area of the bladder neck and urethra is under investigation but no reports of their use in children with neurogenic sphincter incompetence are available (23).

### Procedures to Reconfigure the Bladder Neck

Attempts to induce urinary continence by reconfiguring the bladder neck (BNR) dates back almost 100 years when Young described an operation to correct incontinence in a patient with epispadias (24). The first published application of the Dees' modification (25) of the Young bladder neck reconstruction to patients with neurogenic bladder dates back to 1973 (26). Twelve years later González and Sidi published their experience in 14 patients with neurogenic incontinence treated with a combination of bladder neck reconfiguration, enterocystoplasty (EC) and intermittent catheterization (IC) after a rigorous determination of sphincteric incompetence using a combination of fluoroscopy and electromyography with excellent success in 7 patients. Patients thought to have adequate urethral resistance received only EC. Thirteen of 14 patients became dry (27). Four years later, the same group reported equal degrees of continence with BNR and implantation of an artificial urinary sphincter (AUS) but the complication rate was higher with the AUS (28). This series included patients who had received earlier models of the AUS, known to produce inferior results compared to more modern models. A Canadian group compared BNR in boys with colposuspension in girls. Girls became dry in a greater proportion of cases, however more girls than boys had an EC (29). More recently, Donnahoo et al. reported and initial success of 68% in 38 children with neurogenic incontinence. Ninety-two percent of the patients eventually required EC (30).

In a more recent study, Faure et al. reported on 55 children treated with BNR at a mean age of 7.6 years. Only 10 patients (18%) were considered continent after the isolated BNR and others received additional 2.29 bladder neck injections of a bulking substance. They found no differences in outcome between boys and girls but the results were better in neurogenic patients (54%) than in those with bladder exstrophy (30%) (21).

Other procedures have been described as alternatives to the Young- Dees repair with or without ureteral reimplantation (Ledbetter) (31). Tanagho described the elongation of the urethra with an anterior bladder tube (32, 33) to achieve urinary continence in a variety of conditions but the use of this technique in pediatric neurogenic incontinence has not been reported. Kropp and Angwafo reported a variation of Tanagho's technique creating a tube of the anterior bladder wall implanted submucosally in the midline of the trigone to create a valve mechanism. This technique was designed for children with neurogenic incontinence dependent on IC (34). Salle introduced a modification of this procedure intended to simplify it (35) and published a modification of the original procedure 3 years later (36).

In Kropp and Angwafo initial report 13 children with myelomeningocele reported that all patients stopped wearing diapers and were socially dry with a follow-up between 8 and 36 months (34). Using the same operation Waters et al. (37) reported on 49 patients with NBSD 72% of whom never had difficulty catheterizing per urethra. The problems with CIC occurred both early and late with equal frequency in males and females. The CIC problems were solved by changing the type of catheter and/or avoiding over distension. Two patients with persistent problems required a continent catheterizable channel.

Nakamura et al. (38) reported results of the Salle procedure in 12 children (9 with NSBD) Seven were completely dry (58%) at a mean follow-up time of 75 months. Three had experienced difficulties with urethral catheterization. After repeated procedures all patients became dry but most patients also had EC and a continent catheterizable stoma, indicative of the difficulties with urethral CIC.

Jawaheer and Rangecroft (39) reported results with the Salle procedure in 18 children with a mean follow-up of 24 months. Daytime dryness of 3 h or more was achieved in 61% but 5 remained incontinent. Four children experienced difficulty with urethral catheterization and 39 % required further operations.

Szymanski et al. (40) reported on a group of children who had either the Kropp and Angwafo (*n* = 30) or the Salle (*n* = 8) procedures with mean follow up of 7 and 10 years, respectively. The majority of children also had an EC and an abdominal catheterizable channel. There were no statistically significant differences in the 4 h dry interval between the 2 procedures (Kropp 81.3% and Salle 75.0%) but reoperations were frequent and ultimately most patients did not catheterize urethrally.

### Fascial Slings

Fascial slings operate by compressing the urethra and by elevating the urethra to an intraabdominal position to create resistance and thus increase the passive bladder outlet resistance and leak point pressure. The procedure was initially used to correct female non-neurogenic stress urinary incontinence and patients were expected to continue to void spontaneously. However, the use of slings in NSBD aims at creating an obstruction and spontaneous voiding cannot be expected, therefore clean intermittent catheterization is usually needed.

The first sling procedures were described at the beginning of the last century. McGuire et al. reported the first sling operations in children with NSBD in the 1980s using a rectus fascial sling (41).

Direct comparison of the reported results is limited due to combination of the sling procedure with other procedures (augmentation, BNR), various operation methods and sling materials, patient selection and definition of “continence”. An early series by Barthold et al. reported significantly better results in females than in males with NBSD (42).

In one report, patients reported a better quality of life due to improved continence and longer interval between catheterization when they underwent sling operations with or without bladder augmentation (43).

Various materials have been used to construct the slings including autologous grafts, xenografts, and synthetic materials. In the last years in adult patients synthetic materials have been more widely used. In adolescents Garcia Fernández et al. reported achievement of a dryness interval for at least 3 h in 21/25 patients (84%) with the implantation of a mini-sling (polypropylene mesh with two lateral fixation arms) and only one major complication (44). Nevertheless, in children most reports relate to rectus fascial slings.

In isolated reports, sling implantations have been performed on outpatient basis (45) and with minimally invasive techniques (46). Castellan obtained continence in 51 patients of total 58 patients (88%) with rectus fascial sling procedure and bladder augmentation at follow up at mean 4.1 years. The authors consider the sling procedure as the procedure of choice but they emphasize the necessity of simultaneous bladder augmentation (47).

Snodgrass and Barber (43) reported complete dryness after bladder neck sling in 16 of 35 children (46%) whereas additional of a modified Young-Leadbetter bladder neck procedure (47) improved the results to 14 of 17 (82%) (43). The same group later reported no progressive deterioration in bladder compliance after bladder neck sling operation without augmentation at a mean follow up of 39 months (48). However, recently Noordhoff et al. (49) published the 10 year outcome of 60 patients who underwent bladder neck procedures (43 slings). In the majority of the patients a bladder augmentation (80%) and continent catheterizable urinary channel (97%) were eventually needed. Within 1 year only 15 patients (35 %) were dry and almost half of the children needed additional interventions.

Fascial sling implantations have a low complication rate. Chrzan et al. reported 2 urethral perforations managed conservative treatment in 89 operated children. In their experience, detrusorectomy (50) did not improve the rate of dryness but enterocystoplasty did (51). These authors also suggested that perineal access could help to avoid urethral injury in boys with small operating space or deformity of the pelvis. Dik et al. reported on 24 transvaginal approach to sling implantation in girls with spina bifida, 19 girls were dry after the initial procedure which was sometimes combined with a bladder augmentation or continent stomas. No patient had difficulty with catheterization or infectious complications (52).

### Artificial Urinary Sphincter (AUS)

The results of AUS implantation in children and young adults with neurogenic incontinence from several centers have been published (53). The first implantable AUS was reported in 1973, at a time when CIC was not yet widely accepted. In order to ensure bladder emptying, an external sphincterotomy in males and a Y-V plasty of the bladder neck for females was recommended (54). Since CIC has shown to be compatible with the AUS (55), such emptying enhancing procedures have been abandoned. One may consider separately continence or dryness, the possibility of spontaneous voiding vs. the need for intermittent catheterization, and the need for bladder augmentation. Dryness can be achieved with the AUS in 54 to 100 percent of patients (56–64). Some of the series cited included older models of the sphincter that were not as reliable or durable as the one currently available. If one excludes devices that were removed early because of infection or erosion, the results are even better, around 85% after 5 to 10 years.

Spontaneous voiding in children with NBSD can be expected in 22 to 47 percent, predominantly in patients with spontaneous emptying before implantation (65). However, spontaneous voiding may become difficult after puberty. Replacing the cuff for one of a larger circumference has not restored voiding in these patients (66). For this reason some have recommended waiting till after puberty to implant an AUS (67). From the point of dryness, however, the results are independent of the age of implantation (68).

The most frequent complications of AUS implantation are infection, erosion of structures in contact with the devise (bladder neck, urethra and skin) and mechanical failures. Infections could be minimized with meticulous aseptic technique and erosions by avoiding implantation on areas previously operated and with the new design of the cuff (69, 70). The durability of the AUS has improved significantly since the initial reports (53).

As with all effective means of increasing bladder outlet resistance, a bladder with sufficient capacity and compliance is essential for success and safety. However, even an acceptable bladder may undergo unfavorable changes after the outlet resistance is increased (49, 71). When the bladder capacity and compliance are insufficient, bladder augmentation prior or at the time of AUS implantation has been performed (72, 73). Nevertheless, it is questionable which urodynamic parameters are important to determine the need for augmentation (74, 75). In an attempt to reduce the likelihood of intestinal augmentation related complications others have combined the implantation of AUS with a seromuscular colocystoplasty with more than 85 % dryness at 2 years (76, 77). Others have sought to avoid the potential complications of mechanical malfunction of the AUS by implanting only the cuff at the time of an enterocystoplasty but most patients eventually required implantation of the entire AUS system (78).

### Bladder Neck Closure

Closure of the bladder neck has been reported as a salvage measure when other methods to induce continence have failed. Of course this is only applicable for patients with a good capacity and an alternative to urethral catheterization. Fistulas can occur requiring revision of the closure (79, 80).

## Discussion

The results of this literature review put in evidence a wide variability in results. Nevertheless, it seems clear that little has changed since the 2000 report by Kryger et al. (81) except for a larger number of publications related to bulking agents and slings. The most frequently reported agent is Dx/HA. In general, injection of bulking agents have yielded disappointing results in NSBD as an initial or primary method of treatment. The attractiveness of the simplicity of the method is outweighed by its cost and the very frequent need of repeated injections to obtain at best, modest success. Perhaps the best application of injection of bulking agents is to improve dryness after slings or bladder neck reconstruction.

Bladder neck narrowing procedures, originally reported to correct the anatomy in patients with epispadias has been applied to patients with NSBD. The original Young procedure has suffered several modifications maintaining the principle of elongating the urethra proximally and narrowing the bladder neck. Procedures which create a one way valve, preventing leakage of urine from the bladder but allowing catheterization are more recent. In general the results of all techniques have been equivalent. They share the advantage of avoiding the use of prosthetic materials and being universally available. They all share the disadvantage of reducing bladder capacity, a factor that, added to the well-documented response of the detrusor to obstruction, makes simultaneous bladder augmentation mandatory. Problems with urethral catheterization develop frequently in long term follow up and so it seems wise to combine them with construction of a continent catheterizable channel. We have largely abandoned these operations in neurogenic patients in favor of slings, artificial sphincters or bladder neck closure.

Equally confusing is the literature regarding slings. Since in most reports with high success rates the sling placement has been combined with bladder augmentation and alternative routes for CIC (82), the effectiveness of the sling *per se* is difficult to discern. Only one report showed similar results of slings with and without enterocystoplasty (83). A large part of the problem lies with the difficulties in the preoperative evaluation of the outlet resistance in patients with small and non-compliant bladders (27).

The difference in outcomes between males and females varies also according to the reports (51, 84). In our practice, based on the literature and our own experience, we use slings in females dependent on CIC and largely in combination with a bladder augmentation. We routinely inform patients and families that problems with CIC might arise and that a continent catheterizable channel may be needed in the future.

We continue to implant AUS in males as our preferred method to increase outlet resistance and in females believed to be capable of spontaneous emptying (85). Table 1 summarizes the effectiveness and potential problems of the various treatment modalities.

**Table 1:** Comparison of procedures to increase outlet resistance.

**Procedure**	**Effectiveness**	**Need for augmentation**	**Difficult urethral CIC**	**Need of second procedures**
Injection of bulking agents	7–54%	Unknown	No	Common
Bladder neck reconfiguration	54–68%	100%	Often	Rare if done with augmentation
Slings	36–80%	80%	Often	Common when used alone
AUS	54–100%	30%	No	30%
BN closure	90%	yes	N/A	39%

In our experience we reserve bladder neck closure when other methods have failed and the patient and her/his caregivers understand all the potential risks and potential solutions when catheterization of a full bladder is impossible.

The age at which continence should be achieved in these patients is also a controversial issue. While some emphasize the need to create the expectation of dryness at an early age (85) others propose waiting until after puberty (67).

The surgeon performing procedures to increase bladder outlet resistance assumes a long term commitment to educate and follow these patients given the risks of renal damage and indeed to life if the bladder becomes hostile or patient/caregivers compliance with CIC is not perfect.

## Conclusions

This review of the literature on the methods to increase bladder outlet resistance in patients with NBSD suggest that little progress in obtaining high level of evidence in the last 20 years. Surgeon's experience, personal preferences and open and honest discussion with patients and caregivers are essential to provide the best possible care for these challenging problems.

## Author Contributions

RG and BL conceived the structure of the article. J-CB wrote the section on Slings and bulking agents. RG wrote the majority of the text. AL contributed to the preparation and editing of the manuscript. All authors contributed equally to the final version of the article.

### Conflict of Interest Statement

The authors declare that the research was conducted in the absence of any commercial or financial relationships that could be construed as a potential conflict of interest.
